# Versorgungssituation von CIDP-Patienten in neun deutschen Zentren des Neuritis Netzes

**DOI:** 10.1007/s00115-022-01377-0

**Published:** 2022-08-23

**Authors:** Anna Lena Fisse, Jeremias Motte, Thomas Grüter, Felix Kohle, Cornelius Kronlage, Jan-Hendrik Stahl, Natalie Winter, Tabea Seeliger, Stefan Gingele, Frauke Stascheit, Benjamin Hotter, Juliane Klehmet, Karsten Kummer, Elena K. Enax-Krumova, Dietrich Sturm, Thomas Skripuletz, Jens Schmidt, Min-Suk Yoon, Kalliopi Pitarokoili, Helmar C. Lehmann, Alexander Grimm, Mitglieder des Neuritis Netz, Kalliopi Pitarokoili, Jeremias Motte, Anna Lena Fisse, Thomas Grüter, Juliane Klehmet, Frauke Stascheit, Benjamin Hotter, Min-Suk Yoon, Melis Sevindik, Dilovan Ismael, Helmar Lehmann, Felix Kohle, Martin Svačina, Christian Schneider, Jana Zschüntzsch, Kanan Hasanov, Karsten Kummer, Rachel Zeng, Stefanie Glaubitz, Stefanie Meyer, Karsten Schmidt, Thomas Skripuletz, Stefan Gingele, Tabea Seeliger, Dietrich Sturm, Elena Enax-Krumova, Jens Schmidt, Alexander Grimm, Natalie Winter, Cornelius Kronlage, Jan-Hendrik Stahl

**Affiliations:** 1 Klinik für Neurologie des St. Josef-Hospitals, Katholisches Klinikum Bochum, Universitätsklinikum der Ruhr-Universität Bochum, Gudrunstr. 56, 44791 Bochum, Deutschland; 2 Klinik und Poliklinik für Neurologie, Medizinische Fakultät, Universitätsklinikum Köln, Köln, Deutschland; 3 Klinik für Neurologie mit Schwerpunkt Epileptologie, Hertie-Institut für klinische Hirnforschung, Eberhard-Karls-Universität Tübingen, Tübingen, Deutschland; 4 Klinik für Neurologie mit Klinischer Neurophysiologie, Medizinische Hochschule Hannover, Hannover, Deutschland; 5 Klinik für Neurologie mit Experimenteller Neurologie, Charité Universitätsmedizin Berlin, Berlin, Deutschland; 6 Klinik für Neurologie, Jüdisches Krankenhaus, Berlin, Deutschland; 7 Klinik für Neurologie, Neuromuskuläres Zentrum, Universitätsmedizin Göttingen, Göttingen, Deutschland; 8 Neurologische Universitätsklinik und Poliklinik, BG Universitätsklinikum Bergmannsheil gGmbH Bochum, Ruhr-Universität Bochum, Bochum, Deutschland; 9 Klinik für Neurologie, Agaplesion Bethesda Krankenhaus Wuppertal, Wuppertal, Deutschland; 10 Abteilung Neurologie und Schmerztherapie, Immanuel Klinik Rüdersdorf, Universitätsklinikum der Medizinischen Hochschule Brandenburg Theodor Fontane, Rüdersdorf bei Berlin, Deutschland; 11 Fakultät für Gesundheitswissenschaften Brandenburg, Medizinische Hochschule Brandenburg Theodor Fontane, Rüdersdorf bei Berlin, Deutschland; 12 Klinik für Neurologie, Evangelisches Krankenhaus Hattingen, Hattingen, Deutschland

**Keywords:** Chronisch inflammatorisch demyelinisierende Polyneuropathie, Diagnostik, Diagnosekriterien, Therapie, Patientenversorgung, Chronic inflammatory demyelinating polyneuropathy, Diagnostics, Diagnostic criteria, Treatment, Patient care

## Abstract

**Hintergrund:**

Die Diagnose und Behandlung von Patienten mit immunvermittelten Polyneuropathien ist aufgrund der Heterogenität der Erkrankungen herausfordernd.

**Ziel der Arbeit:**

Ein aktueller epidemiologischer Überblick über die Versorgungssituation von Patienten mit immunvermittelten Polyneuropathien innerhalb des deutschen Neuritis-Netzwerks „Neuritis Netz“.

**Material und Methoden:**

Es erfolgte eine Umfrage in neun deutschen neurologischen Zentren, die auf die Betreuung von Patienten mit Immunneuropathie spezialisiert sind. Wir erfassten Diagnose, Vorgehen in der Diagnostik und Nachsorge, typische Symptome bei Manifestation und im Krankheitsverlauf sowie Therapiedaten.

**Ergebnisse:**

Die Erhebung umfasst Daten von 1529 jährlich behandelten Patienten mit Immunneuropathien, 1320 davon mit chronisch inflammatorisch demyelinisierender Polyneuropathie (CIDP). Die Diagnostik umfasste fast immer Lumbalpunktionen sowie Elektroneuro- und -myografien entsprechend den aktuellen Leitlinien. Der Einsatz von Ultraschall, Biopsie und MRT war unterschiedlich. Wichtigster klinischer Parameter zum Therapiemonitoring in allen Zentren war die motorische Funktion in den klinischen Nachuntersuchungen. Zur Erhaltungstherapie wurde bei rund 15 % der Patienten ein breites Spektrum unterschiedlicher Immunsuppressiva eingesetzt.

**Diskussion:**

Die Studie liefert wichtige epidemiologische Daten zur aktuellen Versorgungsituation von Patienten mit Immunneuropathien in Deutschland. Die Weiterentwicklung spezifischer Empfehlungen zur Therapie und Nachverfolgung von CIDP-Patienten ist notwendig, um einen einheitlichen Standard der Patientenversorgung zu gewährleisten. Dieses wird durch die strukturierte Zusammenarbeit von Exzellenzzentren wie dem deutschen Neuritis Netz erheblich unterstützt.

## Abstract (en)

**Background:**

Diagnosis and treatment of patients with immune-mediated neuropathies is challenging due to the heterogeneity of the diseases.

**Objectives:**

To assess similarities and differences in the current care of patients with immune-mediated polyneuropathies in specialized centers in Germany within the German neuritis network “Neuritis Netz”.

**Material and methods:**

We conducted a cross-sectional survey of nine neurological departments in Germany that specialize in the care of patients with immune-mediated neuropathies. We assessed the diagnosis, the approach to diagnostic work-up and follow-up, typical symptoms at manifestation and progression of the disease, and treatment data.

**Results:**

This report includes data from 1529 patients per year treated for immune-mediated neuropathies, of whom 1320 suffered from chronic inflammatory demyelinating polyneuropathy (CIDP). Diagnostic work-up almost always included nerve conduction studies, electromyography, and lumbar puncture in accordance with current guidelines. The use of ultrasound, biopsy, and MRI varied. The most important clinical parameter for therapy monitoring in all centers was motor function in the clinical follow-up examinations. A wide range of different immunosuppressants was used for maintenance therapy in about 15% of patients.

**Conclusions:**

These data provide important epidemiological insights into the care of patients with immune-mediated neuropathies in Germany. The further development of specific recommendations for treatment and follow-up examinations is necessary to ensure a uniform standard of patient care. This effort is greatly facilitated by a structured collaboration between expert centers such as Neuritis Netz.

## Hinführung zum Thema

Das Spektrum der immunvermittelten Neuropathien ist breit und geprägt von Subtypen, die sich anhand ihrer klinischen, laborchemischen und elektrophysiologischen Eigenschaften teilweise überschneiden. Daher sind Diagnosestellung und Versorgung dieser Patienten herausfordernd. Das Ziel dieser Studie im Rahmen der Kooperation innerhalb des Neuritis Netzes ist es, einen Überblick der aktuellen Versorgungslage von Patienten mit einer Immunneuropathie an insgesamt neun Zentren in Deutschland zu geben.

## Hintergrund

Immunvermittelte Neuropathien sind eine wichtige Differenzialdiagnose peripherer Nervenerkrankungen und stellen ungefähr 10 % aller Polyneuropathien dar [16]. Die Identifizierung und Abgrenzung anderer Ätiologie einer Polyneuropathie ist aufgrund einer sich daraus ergebenden immunmodulierenden Therapie wichtig. Daher erfolgen bei Verdacht umfangreiche diagnostischen Schritte wie Elektroneuro-/-myografie (ENG, EMG), laborchemische Untersuchungen, Lumbalpunktion und ggf. Nerven- und Muskelultraschall, MRT und/oder Nervenbiopsien [16].

Zu den immunvermittelten Neuropathien mit chronischen Verläufen gehören die chronisch-inflammatorische demyelinisierende Polyneuropathie (CIDP) mit ihren Varianten, paraproteinämische Neuropathien, die multifokale motorische Neuropathie, Paranodopathien, Vaskulitiden und Kollagenosen [13, 15].

Aufgrund der Heterogenität der klinischen Charakteristika ist die Diagnosestellung häufig herausfordernd und führt zu einer hohen, zentrumsabhängigen Variabilität [3, 6, 13]. Dies erschwert die Durchführung von randomisiert-kontrollierten Studien und behandelnden Neurologen bleiben oft Therapieoptionen ohne hohe Evidenz [9]. Andererseits fehlen auch für das weitere Monitoring nationale und internationale Guidelines, ebenso wie für Therapieauslassversuche, was zumindest teilweise zu unnötiger und anhaltender immunmodulierender Therapie führt [1, 2, 8].

## Material und Methoden

Es erfolgte eine strukturierte Umfrage mittels eines Fragebogens an die behandelnden Zentren über die Versorgungssituation von Patienten mit Immunneuropathie im Zeitraum von 09/2021 bis 01/2022. Hierbei beteiligten sich neun größere neurologische Zentren (sieben Universitätskliniken und zwei nicht-universitäre Schwerpunkt-Kliniken): Charité Universitätsmedizin Berlin, die Universitätsklinika der Ruhr-Universität Bochum – Katholisches Klinikum Bochum und BG Universitätsklinikum Bergmannsheil Bochum –, Universitätsmedizin Göttingen, Medizinische Hochschule Hannover, Evangelisches Krankenhaus Hattingen, Universitätsklinikum Köln, Universitätsklinikum Tübingen und Agaplesion Bethesda Krankenhaus Wuppertal. Die Beantwortung der Fragebögen erfolgte durch die einzelnen Zentren aus der Erfahrung des klinischen Alltags sowie auf Basis von gegebenenfalls vorhandenen lokalen Registerdaten der Kliniken und Daten erhoben aus dem Abrechnungssystem. Der Umfang des Fragebogens orientierte sich an einer 2020 publizierten Studie von Broers und Kollegen [5]. Die statistische Auswertung und die Erstellung der Grafiken erfolgte anschließend deskriptiv mittels Microsoft Excel und PowerPoint für Mac Version 16.58 (Microsoft Corporation, Redmond, USA).

## Ergebnisse

Insgesamt wurden von allen beteiligten Zentren in einem Jahr über 1500 behandelte Patienten mit Immunneuropathie berichtet, von denen etwa 300 als Neudiagnosen angegeben wurden. Von den 1500 Patienten wurden etwa 1320 als CIDP angegeben, davon etwa 260 als Neudiagnosen. Etwa 60 % dieser CIDP-Patienten befanden sich in Behandlung auf Grund einer typischen CIDP, ca. 40 % aufgrund einer CIDP-Variante definiert nach den Kriterien der European Academy of Neurology (EAN) und der Peripheral Nerve Society (PNS) [17]. Es wurden insgesamt ca. 100 behandelte Patienten mit multifokaler motorischer Neuropathie (davon 16 mit Erstdiagnose), ca. 90 Patienten mit paraproteinämischer Neuropathie (davon 11 mit Erstdiagnose) und ca. 15 mit Paranodopathien (davon 5 mit Erstdiagnose) pro Jahr angegeben.

Erhoben wurden Einschätzungen zu den vorliegenden Symptomen durch die behandelnden Ärzte. Am häufigsten wurde über das Auftreten von sensiblen Symptomen bei ca. 80 % der Patienten berichtet, gefolgt von motorischen Ausfällen bei 65 % und Gangstörungen bei 60 %. Daneben stellten sich Schmerzen bei etwa 40 % und Fatigue bei rund 30 % der Patienten als häufig auftretende Auswirkungen der Erkrankungen dar.

Bezüglich Komorbiditäten wurden Angaben zur Häufigkeit eines Diabetes mellitus und zur Alkoholabhängigkeit gemacht. Die Zentren gaben an, dass ein Diabetes mellitus im Durchschnitt bei ca. 16 % und ein Alkoholabusus bei < 1 % der Patienten mit immunvermittelten Neuropathien bestand.

### Diagnostik bei Diagnosestellung

**Figure 1:**
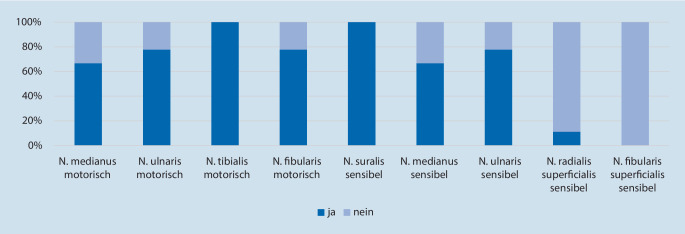

Die beteiligten neun Zentren wurden zur üblicherweise im Rahmen der Abklärung einer CIDP durchgeführten Diagnostik befragt. Der Umfang der routinemäßig durchgeführten ENG nach Angaben der jeweiligen Zentren unterschied sich: von vier motorischen Nerven wurde nur der N. tibialis an allen Zentren routinemäßig untersucht, der N. medianus hingegen nur in sechs von neun, der N. ulnaris und der N. fibularis in jeweils sieben von neun. Sensibel wurde der N. suralis an allen Zentren routinemäßig untersucht, der N. medianus in sechs von neun, der N. ulnaris in sieben von neun und der N. radialis superficialis und N. fibularis/peroneus superficialis hingegen kaum (siehe Abb. 1).

**Figure 2:**
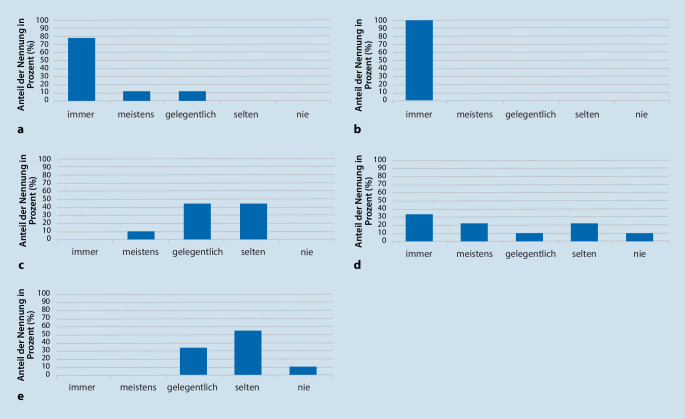

**Figure 3:**
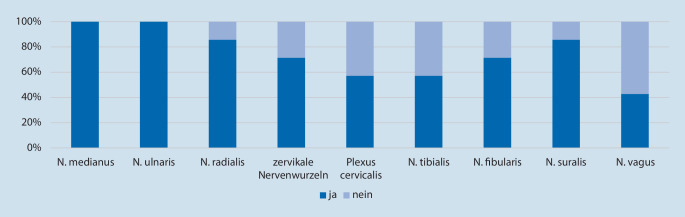

Einen Überblick über die durchgeführte Zusatzdiagnostik gibt Abb. 2. Vom überwiegenden Teil der Zentren (sieben von neun) wurde angegeben, dass immer ein EMG durchgeführt wurde, in zwei Zentren meistens oder gelegentlich. Alle Zentren gaben an, immer Lumbalpunktionen zur Liquoruntersuchung durchzuführen. Ein heterogeneres Bild ergab sich bei der Nervensonografie, die an manchen Kliniken immer durchgeführt wurde, an anderen nie. Der Umfang der Nervensonografie wurde ebenfalls unterschiedlich angegeben (siehe Abb. 3): Nn. medianus und ulnaris gehörten in allen Zentren zur Standarduntersuchung, die Darstellung des Plexus cervicalis und N. vagus erfolgte nur an einigen Zentren. Die MR-Neurografie als weitere bildgebende Untersuchung wurde hingegen insgesamt selten als diagnostisches Mittel herangezogen. Die beteiligten Zentren gaben Schätzungen zur Zahl der jährlich durchgeführten Nervenbiopsien ab. Die Indikation für Nervenbiopsien wurde überwiegend gelegentlich bis selten gestellt. Die Anzahl der Biopsien pro Zentrum variierte mit 5 bis 200 pro Jahr sehr stark und betrug insgesamt ca. 355 in dem Evaluationszeitraum von einem Jahr. Von allen Patienten mit Erstdiagnose einer Immunneuropathie erhielten ca. 64 % eine Nervenbiopsie. Hauptindikation war hierbei der V. a. eine Neuritis in ca. 88 % der Fälle, mit histopathologischer Bestätigung bei ca. 36 % der Patienten.

### Diagnostik zur Verlaufskontrolle

Nach Angabe der beteiligten Zentren erfolgten klinische Verlaufskontrollen von CIDP-Patienten in unterschiedlichen zeitlichen Abständen, meist drei- und sechs-monatlich, in Extremfällen monatlich bis zu zweijährlich (je nach Verlauf). Um eine Beurteilung der Gewichtung der einzelnen Symptome bei klinischen Verlaufskontrollen vornehmen zu können, erfolgte eine Abfrage zur Relevanz hinsichtlich der Verlaufsbeurteilung unter Verwendung einer Bewertungsskala von 1 (= große Wichtigkeit) bis 5 (= keine Relevanz). Motorischen Ausfällen wurde in den befragten Zentren für die Beurteilung der Krankheitsaktivität größte Bedeutung zugeschrieben (Median = 1), wohingegen Fatigue (Median = 5) als nicht relevant bewertet wurde (siehe Tab. 1).

**Table 1:** 

Motorische Ausfälle	1 (1;1)
Gangstörung	2 (2;2)
Schmerzen	3 (3;3)
Sensible Störungen	3 (3;4)
Fatigue	5 (5;5)

**Figure 4:**
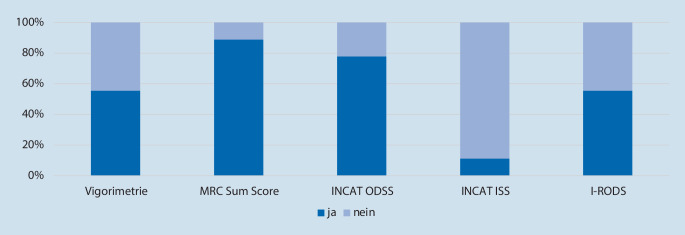

Zur Verlaufsbeurteilung wurde der Einsatz standardisierter Instrumente in verschiedenem Umfang angegeben. Die meisten Zentren gaben an, hierfür prädefinierte Scores und Umfragetools zu nutzen, am häufigsten den Medical Research Council (MRC) Sum Score und den Inflammatory Neuropathy Cause and Treatment Overall Disability Severity Score (INCAT ODSS, siehe Abb. 4).

Die zeitlichen Abstände zwischen elektrophysiologischen und nervensonografischen Verlaufskontrollen bzw. der Umfang solcher Kontrollen wurden als sehr variabel berichtet.

### Therapie

Die Zentren gaben an, dass durchschnittlich 95 % der CIDP-Patienten First-Line-Therapien erhielten (als alleinige Therapie oder in Kombination mit anderen Medikamenten). Von den teilnehmenden Zentren setzten acht von neun üblicherweise Glukokortikoide und sechs von neun intravenöse Immunglobuline in der Ersttherapie ein. In Einzelfällen wurden bei schwerem Beginn in der Ersttherapie Plasmaaustauschverfahren oder eine Immunsuppression mit Cyclophosphamid oder Rituximab angegeben.

Bei fehlendem therapeutischem Ansprechen der Ersttherapie in der Verlaufskontrolle wurde ein Wechsel zur Zweittherapie in vier von neun Zentren auf Glukokortikoide, in vier von neun Zentren auf Plasmaaustauschverfahren, sechs von neun Zentren auf intravenöse Immunglobuline und zwei von neun Zentren auf Immunsuppressiva wie Cyclophosphamid, Azathioprin, Mycophenolat-Mofetil und Rituximab angegeben.

Bei fehlendem Ansprechen der Erst- und Zweittherapie wurden in absteigender Reihenfolge in acht von neun Zentren der Einsatz einer Immunsuppression mit Cyclophosphamid oder Rituximab, in drei von neun Zentren Plasmaaustauschverfahren und in zwei von neun Zentren eine Kombinationstherapie berichtet. Intravenöse Immunglobuline und Glukokortikoide spielten mit jeweils einem von neun Zentren in dieser Situation nur noch eine untergeordnete Rolle. Bei Plasmaaustauschverfahren gaben die Zentren im Durchschnitt an, in ca. 70 % Plasmaseparation und in 30 % Immunadsorption zu verwenden. Der Einsatz von Immunsuppressiva erfolgte bei Auswertung unabhängig vom Krankheitszeitpunkt am häufigsten in Form von Azathioprin (in acht von neun Zentren), Mycophenolat-Mofetil (in sieben von neun Zentren) und Rituximab (in sechs von neun Zentren). Nachgeordnet kamen Cyclophosphamid in vier von neun Zentren, Ciclosporin A in zwei von neun Zentren und jeweils in einem von neun Zentren Methotrexat und Bortezomib zum Einsatz. Insgesamt wurde zur Erhaltungstherapie bei rund 15 % der Patienten ein breites Spektrum unterschiedlicher Immunsuppressiva eingesetzt.

**Figure 5:**
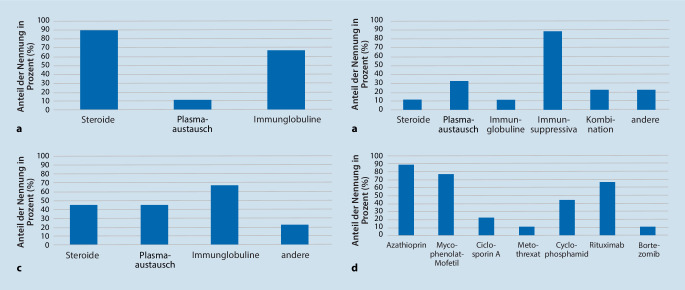

Einen Überblick über die eingesetzten Therapien gibt Abb. 5.

In fast allen teilnehmenden Zentren (acht von neun) wurden Versuche der Dosis- oder Frequenzreduktion von Glukokortikoiden und intravenösen Immunglobulinen angegeben. Veränderungen der Erstlinientherapie wurden zwischen nach vier erfolgten Infusionen (in einem Zentrum) bis nach 6–12 Monaten (in sieben von neun Zentren) angegeben.

Ein ähnliches Bild zeichnet sich bei der Pausierung der Erstlinientherapie ab. In sieben von neun Zentren wurde eine Pausierung nach 6–24 Monaten in Erwägung gezogen, in einem Zentrum bereits nach vier Infusionszyklen. Etwas heterogener war das Bild mit Blick auf die Eskalationstherapie mit Immunsuppressiva. Die allermeisten Zentren (sechs von neun) gaben an, einen Absetzversuch in einem Zeitfenster von 12–24 Monaten zu erwägen, ein Zentrum in einem Zeitfenster von 6–12 Monaten. Ein Zentrum gab an, kaum einen Absetzversuch durchzuführen, ein anderes Zentrum verfolgte keine einheitliche Strategie hinsichtlich eines Absetzversuchs.

Lebensstilveränderungen wurden den CIDP-Patienten teilweise nahegelegt. Fünf von neun Zentren nannten eine Veränderung des Lebensstils als Empfehlung. Dabei wurde die Alkoholkarenz in fünf von neun Zentren am häufigsten genannt, gefolgt von der Empfehlung zur Einnahme von Vitaminpräparaten/Nahrungsergänzungsmitteln in vier von neun Zentren, einer Ernährungsumstellung in drei und einer Nikotinkarenz in zwei von neun Zentren.

## Diskussion

Insgesamt bestätigt unsere Umfrage die bisherige Vermutung, dass ein standardisiertes Vorgehen in Deutschland zur Evaluierung von Immunneuropathien, insbesondere CIDP, nicht gegeben ist. Zwar gaben alle beteiligten Zentren an, bei der Abklärung einer CIDP immer eine Lumbalpunktion durchzuführen, allerdings dient dies neben dem Nachweis einer Blut-Liquor-Schrankenfunktionsstörung bei normaler Zellzahl als unterstützendes Diagnosekriterium in erster Linie dem Ausschluss von Differenzialdiagnosen. Im Vergleich sahen in einer Umfrage unter niederländischen Neurologen von 2020 nur 55 % die Liquordiagnostik als essenziell an [5]. Zudem wird in der aktuellen Revision der EAN/PNS-Empfehlungen [17] vorgeschlagen, unter bestimmten Umständen bei bereits vollständig erfüllten Diagnosekriterien eine Lumbalpunktion nicht durchzuführen. Hingegen sind ausführliche ENG zentraler Bestandteil der internationalen Konsensus-Diagnosekriterien, sodass eher überrascht, dass beispielsweise die routinemäßig durchgeführte ENG in den an dieser Umfrage beteiligten Kliniken eine sehr heterogene Auswahl der untersuchten Nerven ergab. Spezialisierte Untersuchungen wie Nerven-MRT oder Nervenultraschall werden sowohl an den hier beteiligten Zentren wie auch in der Literatur selten berichtet [5], obwohl sie rezent in den neuen EAN/PNS-Richtlinien eine Stärkung erhielten. Auch Nervenbiopsien nehmen in der Diagnostik der CIDP einen mittlerweile geringeren Stellenwert als früher ein.

In den EFNS/PNS-Diagnosekriterien von 2010 [18] und noch expliziter in den aktualisierten EAN/PNS-Empfehlungen von 2021 [11, 17] wird als supportives Diagnosekriterium ein „objektives“ Therapieansprechen gefordert, während eine subjektive Besserung auf Immuntherapie wie intravenöse Immunglobuline oder Steroide auch in CIDP-Fehldiagnosen häufig berichtet wird [2]. Der Einsatz standardisierter Skalen (wie INCAT, RODS) oder objektiver klinischer Messungen (wie Handkraft-Vigorimetrie) zur Verlaufskontrolle und Beurteilung des Therapieansprechens wurde von den in dieser Umfrage beteiligten Zentren in den meisten, aber nicht allen Fällen angegeben.

Für die behandelnden Ärzte sind motorische Störungen in der Verlaufsbeurteilung das wichtigste Kriterium, hingegen gehören sensible Störungen, Schmerzen und Fatigue zu den von Patienten berichteten am stärksten im Alltag die Lebensqualität einschränkenden Symptomen [14]. Apparative Untersuchungen zur Verlaufskontrolle der CIDP werden in unterschiedlicher Frequenz und Umfang durchgeführt. Sowohl für die Elektrophysiologie [4, 7] als auch für die Nervensonografie [10, 12, 19] gibt es Hinweise auf eine Korrelation mit dem Therapieansprechen und prognostische Aussagekraft, wobei dies nicht Einzug in die aktuellen 2021 EAN/PNS-Kriterien gefunden hat.

Die Erstlinientherapie der befragten Zentren ist mit Glukokortikoiden und Immunglobulinen relativ einheitlich und den Leitlinien entsprechend [17], wobei bei der Auswahl der Erstlinientherapie keine einheitlichen Strategien zur Entscheidung für oder gegen eines der beiden Medikamente existieren. Die in der Eskalationstherapie eingesetzten Immuntherapien sind umso heterogener.

Die Stärke der vorliegenden Arbeit liegt darin, dass sie die klinische Versorgungssituation von CIDP-Patienten an einer nennenswerten Zahl spezialisierter neurologischer Zentren abbildet und in Bezug zur wissenschaftlichen Evidenz und Empfehlungslage setzt. Die überwiegende Beteiligung großer, universitärer Kliniken an der Umfrage stellt andererseits eine Limitation hinsichtlich Generalisierbarkeit auf die Versorgungssituation in Deutschland insgesamt dar. Außerdem ist das Neuritis Netz ein offener Zusammenschluss mehrerer interessierter Kliniken, welches allerdings nicht alle auf Immunneuropathien spezialisierten Zentren in Deutschland umfasst. Innerhalb des Neuritis Netzes werden einheitlich die EAN/PNS-Kriterien[17] für die Diagnose der CIDP verwendet. Eine zusätzliche Kontrolle der korrekten Anwendung der diagnostischen Kriterien wurde nicht durchgeführt. Ferner wurde in den Fragebögen die allgemeine Vorgehensweise („in der Regel …“) in den verschiedenen Abteilungen abgefragt, die sich von der klinischen Praxis in atypischen Fällen unterscheiden könnte. Zuletzt handelt es sich um eine Umfrage, bei der von beteiligten Zentren angegebene Fallzahlen und Häufigkeiten teils geschätzt wurden – eine höhere Datenqualität würde in einem multizentrischen Register erreicht, wie es von den Autoren vorbereitet wird.

## Fazit für die Praxis

Die Versorgungssituation der CIDP Patienten in der Praxis ist trotz der 2021 aktualisierten und konkretisierten EAN/PNS-Empfehlungen heterogen. Die derzeitige wissenschaftliche Evidenz zur Diagnostik, Verlaufsbeurteilung und insbesondere zur längerfristigen Therapie von Patienten mit immunvermittelten Neuropathien ist unvollständig. Das deutsche Neuritis Netz hat sich zur Aufgabe gemacht, die Versorgung von Betroffenen mit Neuritis wie der CIDP zu verbessern und wissenschaftliche Netzwerkaktivitäten zu ermöglichen.
